# International Collaboration on Air Pollution and Pregnancy Outcomes (ICAPPO)

**DOI:** 10.3390/ijerph7062638

**Published:** 2010-06-17

**Authors:** Tracey J. Woodruff, Jennifer D. Parker, Kate Adams, Michelle L. Bell, Ulrike Gehring, Svetlana Glinianaia, Eun-Hee Ha, Bin Jalaludin, Rémy Slama

**Affiliations:** 1 UCSF, Program on Reproductive Health and Environment, Oakland, CA 94612, USA; 2 National Center for Health Statistics/CDC, Hyattsville, MD 20782, USA; E-Mail: jdp3@cdc.gov; 3 Health Effects Institute, Boston, MA 02110, USA; E-Mail: kadams@healtheffects.org; 4 Yale University, New Haven, CT 06511, USA; E-Mail: michelle.bell@yale.edu; 5 IRAS, Utrecht University, Utrecht, 3508 TD, The Netherlands; E-Mail: u.gehring@uu.nl; 6 Newcastle University, Newcastle, NE2 4AX, UK; E-Mail: svetlana.glinianaia@ncl.ac.uk; 7 Ewha Womans University, Seoul 158–710, Korea; E-Mail: eunheeha@ewha.ac.kr; 8 University of New South Wales, Sydney 2052, Australia; E-Mail: b.jalaludin@unsw.edu.au; 9 Inserm, Institut National de la Santé et de la Recherche Médicale, U823 Institut Albert Bonniot, Avenir Team “Environmental Epidemiology Applied to Fecundity and Reproduction”, Grenoble, La Tronche, France; E-Mail: remy.slama@ujf-grenoble.fr

**Keywords:** air pollution, pregnancy outcomes, low birthweight, preterm birth, particulate matter, ozone, carbon monoxide

## Abstract

Reviews find a likely adverse effect of air pollution on perinatal outcomes, but variation of findings hinders the ability to incorporate the research into policy. The International Collaboration on Air Pollution and Pregnancy Outcomes (ICAPPO) was formed to better understand relationships between air pollution and adverse birth outcomes through standardized parallel analyses in datasets from different countries. A planning group with 10 members from 6 countries was formed to coordinate the project. Collaboration participants have datasets with air pollution values and birth outcomes. Eighteen research groups with data for approximately 20 locations in Asia, Australia, Europe, North America, and South America are participating, with most participating in an initial pilot study. Datasets generally cover the 1990s. Number of births is generally in the hundreds of thousands, but ranges from around 1,000 to about one million. Almost all participants have some measure of particulate matter, and most have ozone, nitrogen dioxide, sulfur dioxide and carbon monoxide. Strong enthusiasm for participating and a geographically-diverse range of participants should lead to understanding uncertainties about the role of air pollution in perinatal outcomes and provide decision-makers with better tools to account for pregnancy outcomes in air pollution policies.

## 1. Introduction

Numerous studies have investigated associations between outdoor air pollution and perinatal health outcomes, including low birth weight, preterm delivery, and infant mortality [1–5]. These studies provide accumulating evidence for including perinatal outcomes in future national and international (WHO) reviews of air quality standards that previously have little considered these outcomes. Recent qualitative syntheses of these studies have concluded that there is likely an adverse effect of air pollution on pregnancy outcomes [6]. However, there is substantial inconsistency in the methods and findings of these studies, hampering efforts to synthesize the existing evidence, in particular in the form of a meta-analysis. Differences among results include the pollutants associated with the adverse pregnancy outcome and the exposure window of concern. Inconsistencies in the findings among studies may arise from many aspects of each study’s design. Exposure definitions (including methods for assessment, time-periods of exposure, spatial resolution, available pollutants, and combinations of pollutants), outcome definitions, and use of potential confounders and effect modifiers often differ among studies. The differences in study design and lack of consistency across study results hinders the ability of decision-makers to incorporate the research evidence into policy and also slows the pace of scientific discovery of how air pollution impacts pregnancy outcomes. Scientific reviews of the evidence recommended additional research on this topic [1–5].

Two international workshops of experts in air pollution and pregnancy outcomes were held in 2007, in Munich, Germany and Mexico City, Mexico, in which the strengths and limitations of the existing literature were discussed. Although identifying the limitations most likely to play a role in the existing literature is a complex task, the workshops identified confounding and exposure misclassification as points of particular concern [7,8]. One of the key recommendations from the Mexico City workshop was to develop an international collaboration among researchers in the field to apply the same or similar methods to analyzing the existing datasets. Applying a consistent analytic strategy across many datasets may help to reconcile some of the apparent inconsistencies in effect estimates observed across studies. Furthermore, this type of research synthesis would help guide policy. The role of different analytic strategies contributing to heterogeneity in air pollution epidemiology has been previously recognized for adult mortality, and a similar project was conducted with datasets in Europe, United States and Canada under APHENA (Air Pollution and Health: A Combined European and North American Approach) [9].

A focused research effort is critical in reproductive health as outdoor air pollution is an increasingly important global health hazard, with growing industry and vehicle use in many parts of the world and with potential impacts on vulnerable populations, such as the developing fetus. Thus, there is a growing focus on the reproductive implications of air pollution in many areas of the world, as demonstrated by the European Environment and Health Strategy development of the Children’s Environment and Health Action Plan for Europe (CEHAPE) [10]. Further, understanding harmful prenatal exposures is a critical area of research need with broad public health implications. In addition to immediate consequences, adverse birth outcomes such as preterm delivery and low birthweight, may increase the risk of later onset of adult disorders, such as diabetes and cardiovascular disease [11,12].

The International Collaboration on Air Pollution and Pregnancy Outcomes (ICAPPO), was formed, based on a recommendation from the Mexico City workshop [8], to develop and conduct analyses, using a standardized methodology across multiple investigator generated datasets from different study settings, to gain insights into how design options and analytic decisions affect results from perinatal environmental epidemiological studies and, potentially, to provide comparable results for research synthesis. We hypothesize that some of the differences among the published results of pregnancy air pollution exposure studies can be attributed to identifiable differences in analytic methodologies, including the composition of the study populations, exposure assignments, and availability and use of covariates. In addition, standardizing the analytic strategies among the studies will enhance our ability to conduct a meta-analysis, and will provide a more robust result than combine the results of the currently independently conducted studies.

A third international workshop was held in 2008 in Pasadena, USA, to propose a way forward for ICAPPO. This workshop included planning for a collaborative pilot study and discussing epidemiologic methods, particularly those that can be applied in different settings, for collaborative analyses. A fourth workshop was held in 2009 in Dublin, Ireland to hone the study’s aims and discuss future directions.

This paper describes the formation of the ICAPPO, identifies and lists its aims, and reviews the participating study locations, including study design, exposure metrics, and available outcome and covariate information.

## 2. Methods

### 2.1. Establishment of the ICAPPO

The ICAPPO was established to coordinate an international effort toward understanding how much differences in methodology contribute to variation in study findings. Most ICAPPO participants are researchers in the field of air pollution and pregnancy outcomes who already have datasets that link maternal exposure to air pollution with pregnancy outcomes; many of these participants have published previous papers on this topic, which comprise the major papers in the field. Other ICAPPO participants do not have an existing data source available but have expressed an interest in participating. While many ICAPPO participants are those actively involved in the research goals of this project, other researchers have provided significant contributions to the overall aims of the ICAPPO through their insights during the workshops. Currently, participation is an open process and there are no dataset criteria to participate. We identified possible participants based on publications in the peer-reviewed literature and recommendations from other researchers.

The primary aim of ICAPPO is data re-analysis, so participants are asked to reanalyze or recalculate data in certain ways, we are not asking for new data collection or new variables. Participation in the ICAPPO does not in any way hinder researchers’ ability to conduct other studies with their datasets.

As part of the collaboration, we have facilitated collecting information about the eligible datasets, primarily through review and posting on a wiki hosted as part of ICAPPO. This information has been and will be used to guide protocols for re-analysis which can take advantage of the common features and distinctions of the existing data. Participants all have access to the wiki, and other researchers who are interested in the topic can also have access by emailing prhe@obgyn.ucsf.edu.

### 2.2. Collected Information on Individual Study Attributes

The first step for the ICAPPO was to collect information on the data attributes from each of the study locations with respect to the availability of variables, timeframe, study location, and other factors. Participants were asked to provide the following information: location, available air pollutants, study period, number of births included in the study (or number of study subjects), and covariates available (e.g., mother’s smoking status during pregnancy, gestational age, mother’s race). Mostly these datasets have been used for previously published studies, are currently being used for ongoing studies, or both. Datasets have typically been constructed from some type of air pollution exposure metric linked to data available from routinely collected administrative records (birth certificates), birth record datasets constructed for a specific study, or to data from a pregnancy or birth cohort study. Because each dataset’s construction was tied to a variety of independent research questions unrelated to ICAPPO, there is variability in how the datasets have been or are being developed and the specifics of each study’s design. For example, many studies have used air pollution monitors to assess maternal exposures, while some studies use other methods, such as land-use regression or other modeling techniques.

### 2.3. Research Aims

ICAPPO participants are working together to prioritize the research questions for the analytical protocols. Research questions were identified through previous workshops (Munich and Mexico City) and were honed at workshops in Pasadena, California USA, and Dublin, Ireland. The primary areas of focus include:

- To evaluate whether different exposure metrics, and/or approaches used to define exposure to air pollutants, influence the relationship between air pollution and pregnancy outcomes.
- To identify how the consideration of covariates affects the relationship between air pollution and pregnancy outcomes. In particular, we will focus on previously identified covariates that may influence the relationship between air pollution and pregnancy outcomes; we will consider covariates measured at the individual level (e.g., maternal age) and at the area level. Area-level covariates will include both socio-demographic indicators (e.g., urbanicity) and physical indicators including temperature, season, and co-pollutants.
- To investigate methods for considering critical periods of susceptibility during pregnancy (whole pregnancy, trimesters, and months of pregnancy); often exposures during these periods are correlated and decisions about which time periods to study and how to consider possible correlation may affect inferences.
- To identify whether season of exposure influences any observed relationships between air pollution and pregnancy outcomes.
- To investigate the effects of exposure to co-pollutants on the strength of the relationship between pregnancy outcomes and individual pollutants.

Although a small group of researchers from ICAPPO will develop methodological approaches for each focus area, all participants will have input into the methods. Due to the inherent differences among the datasets—for example, not all datasets have data for all pollutants or covariates of interest—not all datasets can be involved in every focus area of the project. Each location will carry out analyses for their own dataset.

Results from these new analyses will be evaluated for variations, and potential reasons for the variations, in the associations between outdoor air pollution and pregnancy outcomes. Results will be evaluated across the study locations using graphical and statistical techniques for understanding variability in the findings. Our overall goal is to be able to combine the results from the parallel projects to examine factors that affect differences among the study locations and, if appropriate, to develop combined estimates of association, through meta-analysis techniques.

### 2.4. Pilot Study

An initial first step in the collaboration was to conduct a pilot study. The aims of the pilot study are to test the dynamics of ICAPPO, the practicalities of conducting parallel analyses across multiple sites, and the feasibility of conducting a pooled analysis. The pilot study, currently in progress, investigates the effects of exposure to PM10 during pregnancy on low birth weight (<2,500 g) in term live births (37–42 weeks of gestation). Since an objective of the pilot study was to maximize the number of participating research groups, we chose PM10 as it was the air pollutant most often analyzed, and one birth outcome, low birth weight in term births, as this was most easily accessible by most researchers and comparable across study locations. Socioeconomic status is included as a potential confounder. A protocol for the pilot study was developed by a sub-team of ICAPPO with input from all participants, and then distributed to all participants. Parallel analyses for the pilot study have been or are being conducted by researchers in a number of different centers located across the globe. After the pilot study is completed, the results will then inform the structure of ICAPPO and the next stage in the analytical protocol.

## 3. Results

**Figure 1.**
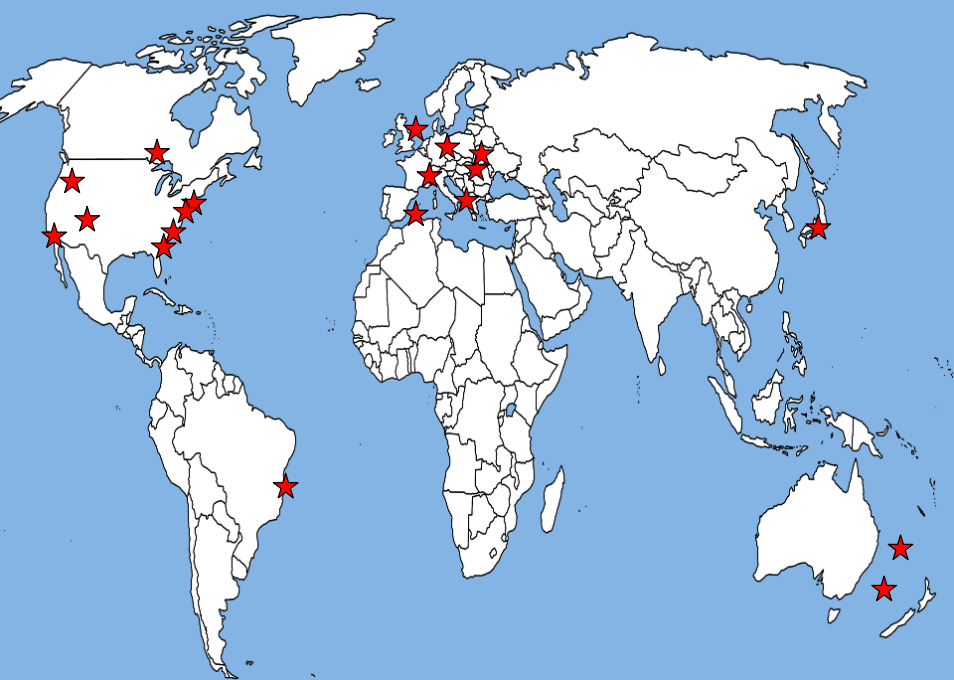
Map of the study locations of the datasets part of the International Collaboration on Air Pollution and Pregnancy Outcomes.

Currently, the ICAPPO has information from over 20 separate research groups in North America, Europe, Australia, Latin America and Asia. Figure 1 and the Table 1 describe the study locations. The number of births that are available per dataset in each study location is generally in the range of 50,000 to 500,000, though there are smaller and larger studies (Table 1). Several of the studies have been recently identified and we are still working on collecting some of the information, so the information in the Table continues to be updated.

In general, the birth years in each study location span the years of the mid 1990s to about 2005 (one study has estimated exposure data back to 1962 (Newcastle upon Tyne, Northeast England) (Table 1). Most studies rely on birth certificate data for the pregnancy outcomes, and a few of the studies are cohorts of women who were surveyed during pregnancy. The Table shows the air pollutants available at each location. The datasets for every study location have some measurement of particulate matter (PM) (19 have PM_10_ data and 15 have PM_2.5_ data), and most of the study locations have measurements for other common pollutants, including ozone, carbon monoxide, sulfur dioxide, and nitrogen dioxide. To estimate exposures, some study locations use measurements from the nearest air pollution monitor, with ranges from 2 km up to 10 km, some locations use averages of measurements from multiple monitors over geopolitical units, such as counties in the United States. Other studies have metrics based on distance to traffic sources or individual exposure estimates from exposure models. A few of the study locations have additional air pollutant data, such information on hazardous air pollutants (a group of air pollutants defined in the US which are separate from those evaluated in these studies) and lead.

The available datasets primarily link pregnancy outcome data to some measurement of air pollution data. Most data on pregnancy outcomes stem from birth certificates; thus, information on the birth outcomes and related covariates is primarily based on what is available from the birth certificates in each location. Information on the key covariates available from each of the study locations is given in Table 2. All study locations had data on gestational age, typically measured through recall of last menstrual period, and birth weight. Most birth certificate data include maternal and infant characteristics, such as education, parity, marital status, age, tobacco and alcohol use (sometimes), prenatal care, residence at birth, gestational age, birth weight, and date of birth (either exact date or year and month). Depending on the original source of the birth certificate data, some data may be aggregated. For example, the exact residence at birth or the exact date of birth may not be known in a public dataset.

The availability of information on race and ethnicity varies, primarily by geographic locations; while race and ethnicity are important predictors of birth outcomes in the United States, other indices, such as immigration status or language are important factors in other countries. Many of the study locations also use one or more community level demographic or socioeconomic indicators (e.g., income levels by census tract or zip code in the USA). A few of the collaborating study locations (Los Angeles, USA; Generation R study, Netherlands; INMA cohort, Spain; Eden cohort, France; and Brisbane, Australia) also have questionnaire data available in addition to birth certificate information, which can be used to evaluate the quality and analytic usefulness of the more widely available covariates (such as detailed smoking information, income level, *etc*.).

## 4. Discussion

We hypothesize that this novel collaboration combining worldwide individual research efforts into an efficient uniform design can provide sufficient important information to answer some of the critical questions in the field of air pollution and perinatal epidemiology. At a minimum, once methodologies are standardized, we will be able to identify factors that contribute to observed remaining variations among the findings from the individual study locations, many of which may be due to variations in site characteristics (such as population differences, source differences for particular pollutants, *etc.*). In addition, once standardized methodological approaches are applied across the datasets, our ability to compare results across locations should improve, including our ability to combine results through meta-analysis.

Challenges of the ICAPPO relate to its ambitious nature. We have enthusiastic response from a wide range of geographic locations, although not all parts of the world are well represented, with participation primarily from North America and Europe. However, covering more areas of the world must be balanced with resources for adding additional collaborators. Although numerous researchers have expressed interest in participating in the study, the logistics of coordinating such a large effort are not trivial. Conducting the pilot study will allow us to evaluate the level of participation in the collaboration and the number of study locations may drop or increase as other commitments increase or new participants and study locations may be added.

Initial collection of dataset information from the study locations indicates that we have a robust number of studies, with information on the primary air pollutants of interest. Given that many of the studies use existing birth certificate data, there is relatively good level of comparable covariate information across the study locations. However, there are still challenges in addressing remaining variability in some of the key covariate data for the analysis. For example, maternal education will be used as the primary proxy for SES. This is a commonly used index of SES in perinatal studies; is correlated, albeit imperfectly, with SES; and is also an important determinant of pregnancy outcomes. While most study locations have some measure of maternal education, there is variability in the construct of the measure, both in type of data used to for the measure (e.g., most have maternal education, a few locations have area level variables) and the cultural context of maternal education (e.g., the percentage of women who complete high school varies from location to location). While we will standardize some of these covariates across data sets, there will still remain some heterogeneity, which may contribute some bias to the interpretation.

In addition, there is some variation in the type of air pollutants available for each location. All the studies have some measure of particulate matter and a reasonable coverage of common gaseous air pollutants. There should be sufficient information to carry out analyses with multiple locations; although not every location will be able to participate as each of the air pollutants are considered. Finally, there will be some challenges when evaluating questions related to geographic scope of the air pollution metrics, as those studies using air pollution monitoring data use varying geographic scales for averaging pollutant exposures. The variation in geographic resolution may be difficult to standardize among the studies, as setting up the metrics involved a number of analytical steps that may difficult to redo/alter.

With ICAPPO, we take a different approach than other efforts such as the EU-funded GA2LEN and ESCAPE projects to gain insights from multiple cohorts. Within the GA2LEN study, data from individual researchers is pooled [13,14]. As part of the ESCAPE project a standardized air pollution exposure assessment is added to a large number of cohort studies [15]. ICAPPO’s approach is to perform parallel investigations with a uniform framework of methods. Results will be compared and potentially pooled after participants apply the standardized study methodologies. The advantage of this approach compared to conducting a new international study of this scale is its efficient use of resources, as collecting data, especially from multiple countries, entails significant administrative and logistical resources. The disadvantage is that it will be difficult to assess the underlying data for comparability/anomalies in the information. This difficulty can be a strength, however, as differences among datasets can be summarized and explored to provide insight into results from individual studies on air pollution and pregnancy outcomes and to inform future research designs. The results of this effort will inform the usefulness of this approach for similar approaches in other related fields.

## 5. Conclusions

ICAPPO will provide important information about the relationship between air pollution and pregnancy outcomes and improve our ability to compare results across numerous geographic locations and studies. It should also greatly enhance our understanding of the effects of air pollution on pregnancy outcomes and motivate focused research questions in the field, such as biological mechanisms that link exposure to specific outcomes. It will also strengthen efforts in individual countries to understand and ultimately mitigate harmful effects from air pollution, by enhancing understanding of their individual place-based study results in the context of comparable results from other study locations around the world. Finally, it will create a network of researchers working across the globe on environmental and pregnancy outcomes generally, which will leverage other opportunities to evaluate how the environment can influence birth outcomes, and ultimately lead to insights that will inform activities to prevent harmful exposures and improve the health of children worldwide.

## Figures and Tables

**Table 1.** Number of births, birth years, and pollutants measured by study location.

Study Location	Birth years	Approximate Number of births	Pollutants measured					
			Carbon Monoxide	Nitrogen Dioxide/Monoxide	Sulfur Dioxide	Ozone	PM10^2^	PM2.5^2^
Atlanta, Georgia, USA	1994–2004	515,000^1^	X	X	X	X	X	X
Brisbane, Australia^4^	2007–2008	960		X	X	X	X	
California, USA	1996–2006	6,000,000^1^	X	X	X	X	X	X
Vancouver, Canada	1999–2002	66,600					X	X
Colorado, USA	1996–2004	572,600	X	X	X	X	X	X
Teplice, Prachatice, Prague, Czech Republic^4^	1994–2002	12,300	X	X	X	X	X	X
Lombardy, Italy		213,500					X	
Los Angeles County, California, USA^4^	2003	58,300	X	X		X	X	X
Los Angeles County, California, USA	1994–1996	48,100	X	X		X	X	
Massachusetts and Connecticut, USA	1999–2002	358,500^1^	X	X	X		X	X
New Jersey, USA	1999–2003	200,000	X	X	X	X	X	X
Newcastle upon Tyne, UK^3^	1962–2002	119,900^1^	X	X	X	X	X	
North East England, UK	1985–2003	665,400^1^	X	X	X	X	X	
North, Center, and West regions, the Netherlands (PIAMA cohort)^4^	1996–1997	3,900		X				X
Poitiers and Nancy (Eden cohort), France^4^	2003–2006	1,900	X	X	X	X	X	X
Lotz, Poland^4^	2007	130					X	X
Ribera d’ Ebre, Menorca, Granada, Valencia, Sabadell, Asturias, and Gipuzkoa, Spain (INMA cohort)^4^	1997–2007	3,900		X		X		X
Rotterdam, the Netherlands, (Generation R cohort^4^	2002–2005	8,880		X		X	X	X
Sao Paolo, Brazil	1997	158,800					X	
Seoul, South Korea*	1996–1998	388,10	X	X	X		X	
Seoul, Pusan, Incheon, Daejeon, Daegu, Ulsan, Kwangju, South Korea*	2004	177,600	X	X	X	X	X	
South Coast Air Basin, California, USA	1994–2000	479,170	X	X		X	X	X
Sydney, Australia	1994–2007	950,000^1^	X	X	X	X	X	X
Virginia, USA	1996–2004	874,100^1^	X	X	X	X	X	X
Washington, USA	1998–2005	294,100						X

1 Not all births have information for all air pollutants

2 Particulate matter is measured in different ways, such as PM_10_, PM_2.5_, sulfates and elemental carbon.

3 For 1962–1992 (about 89,000 births) data are available for black smoke (BS ≈ PM_4_) only

4 Cohort studies, or other interview based data, all other studies linked record information (primarily birth certificate) with air pollution data.

**Table 2.** Availability of Key Covariate Maternal and Infant Covariate Information from each study location.

**Study Location**	**Infant Information**			**Mother Information**				
	Gestational Age	Parity	Sex	SES Indicator	Age	Smoke during pregnancy	Race/ethnicity or country of origin	Marital Status
Atlanta, Georgia, USA	X (LMP)	X	X	Attained education, year	X	X	X	X
Brisbane, Australia	X (LMP)	X	X	Attained education, years	X	X	X	X
California, USA	X (LMP)	X	X	Attained education, years	X	Some	X	Some
Vancouver, Canada	X (LMP)	X	X	Community-level SES	X	X	X	
Colorado, USA	X (LMP)	X	X	Attained education, years	X	X	X	X
Teplice, Prachatice, Prague, Czech Republic	X (LMP)	X	X	Attained education, years	X	X	X	X
Lombardy, Italy	X (LMP)	X	X	Attained education, degree	X		X	X
Los Angeles County, California, USA	X (LMP)	X	X	Attained education, years	X	Inc.	X	X
Massachusetts and Connecticut, USA	X (LMP)	X		Attained education, years	X	X	X	
New Jersey, USA	X (LMP)	X	X	Attained education, years	X	X	X	X
Newcastle upon Tyne, UK	X (LMP)	X	X	Community-level SES; Townsend score	X			X
North East England, UK	X (LMP)	X	X	Community-level SES; Townsend score	X			
North, Center, and West regions, Netherlands (PIAMA cohort^4^	X (LMP)	X	X	Attained education, degree	X	X	X	
Poitiers and Nancy, France (Eden cohort)	X (LMP)	X	X	Age at completion of education	X	X	X	X
Lotz, Poland ^4^	X (LMP)	X	X	Attained education, degree	X	X		X
Ribera d’ Ebre, Menorca, Granada, Valencia, Sabadell, Asturias, and Gipuzkoa, Spain (INMA cohort)	X (LMP)	X	X	Attained education, years	X	X	X	X
Rotterdam, the Netherlands, (Generation R cohort)	X (LMP)	X	X	Attained education, years	X	X	X	X
Sao Paolo, Brazil	X (LMP)	X	X	Attained education, years	X		X	X
South Korea	X (LMP and ultrasound	X	X	Attained education, years	X			X
Sydney, Australia	X (LMP)	X	X	Socioeconomic disadvantage; area-level indicator	X	X	X	X

Virginia, USA	X (LMP)	X	X	Attained education, years	X	X	X	X
Washington, USA	X (LMP)	X	X	Attained education, years	X	X	X	X
